# A Case of the Serotonin Syndrome Secondary to Phenelzine Monotherapy at Therapeutic Dosing

**DOI:** 10.1155/2015/931963

**Published:** 2015-03-16

**Authors:** A. Malik, N. Junglee

**Affiliations:** ^1^Morriston Hospital, Swansea, Wales SA6 6NL, UK; ^2^University Hospital of Wales, Cardiff, Wales CF14 4XW, UK

## Abstract

A 27-year-old Caucasian female with a history of depression was admitted to our local hospital with vague events that occurred a day before. This included an episode of dysarthria, and unsteadiness, followed by feeling generally unwell. Two weeks prior to presentation she was commenced on phenelzine. During clinical assessment she suddenly deteriorated with a dramatic fall in her conscious level. Moreover, she became hyperthermic, tachycardic, and diaphoretic, and developed increased tone in all muscles and ocular clonus. Rectal diazepam was administered but failed to control the symptoms. Consequently, she was transferred to the intensive care unit for intubation and muscle relaxants were commenced. She responded well and recovered next day without complications. Her symptoms and signs were consistent with the serotonin syndrome with phenelzine being the likely cause. To the best of our knowledge, this is the first reported case to associate the condition with phenelzine alone at therapeutic dose.

## 1. Background

The serotonin syndrome is an underreported, underrecognised, and potentially life-threatening iatrogenic condition that occurs on administration of medications that are known to increase the postsynaptic concentrations of serotonin (5-hydroxytryptamine). It is seen with therapeutic medication use, inadvertent interactions between drugs, and intentional self-poisoning. The incidence of serotonin syndrome is unknown as manifestations may be wrongly attributed to another cause, mild cases may be dismissed, or clinicians may not suspect the condition. It is well recognised that presentation is variable; hence diagnosis is challenging. However, owing to the increased use of serotonergic medications the prevalence and therefore clinical importance of serotonin syndrome are likely to increase [1]. This case report demonstrates a case of the serotonin syndrome secondary to monotherapy with phenelzine, an irreversible monoamine oxidase (MAO) inhibitor, and illustrates the importance of swift recognition and management to ensure a favourable outcome. To the best of our knowledge, this is the first reported case to associate phenelzine with the condition.

## 2. Case Presentation

A 27-year-old Caucasian female was admitted to hospital by her family doctor with a one-day history of unsteadiness and feeling generally unwell. This was preceded by a five-minute episode of dysarthria which spontaneously resolved without lasting deficit. Several hours later, she experienced unsteadiness whilst on her feet, describing it as if she were drunk. Subsequently, the patient visited her family doctor the following day and was sent to the local hospital for further assessment in view of the above events. Her history was corroborated by her partner.

Upon arrival the patient felt well apart from a mild headache. She was able to give a history and denied any other symptoms. Systemic enquiry was unremarkable. Her past medical history included depression, suspected epilepsy with normal investigations, and episodic migraine since the age of nine. Her medications on admission included folic acid 5 mg od PO, phenelzine 15 mg tds PO started two weeks prior to admission, and propranolol 40 mg bd PO. She denied consumption of tyramine-rich foods whilst taking phenelzine. On further inquiry she was noted to be on mirtazapine (an atypical antidepressant) before phenelzine and mirtazapine were stopped two weeks before she commenced phenelzine. Prior to this period she was taking sertraline which stopped in June 2010. Her psychiatric disease was generally stable. There were no known drug allergies or side effects experienced with mirtazapine or sertraline. She was a nonsmoker and denied alcohol intake or illicit drug use. There was no history of drug overdose. She lived with her partner and two children.

On examination, she was orientated to time, place, and person. Her GCS was 15. Vital observations included a tympanic temperature of 36.9 degrees centigrade, radial pulse of 80 per minute, and regular blood pressure of 100/54 mmHg sitting, and oxygen saturations at 95% on air with a respiratory rate of 18 per minute. The only positive finding on systemic examination was dilated pupils, 5 mm round and reactive to light.

However, twenty minutes after initial assessment, her GCS dramatically deteriorated to 9 (E4, M4, and V1). Her temperature increased from 36.9 to 40.4 degrees centigrade. She also became diaphoretic and tachycardic at 164 per minute (electrocardiogram confirmed sinus tachycardia). Neurological examination now revealed increased tone (including clonus) and brisk reflexes throughout. Babinski's sign was positive bilaterally. Although her pupils remained dilated and reactive to light, ocular oscillations were apparent but fundoscopy was normal. An arterial blood gas on 10 L/min of oxygen revealed pH 7.45 (7.35–7.45), pCO_2_ 4.56 kPa (4.66–6.0), p02 24.9 kPa (10.6–15.3), bicarbonate 23.4 mmol/L (22–30), and base excess 0.1 mmol/L (−2.0–2.0).

## 3. Investigations

Initial blood investigations were as follows: haemoglobin 12.1 g/dL (4–11), white blood cell count 15 × 109/L (4.0–11.0), neutrophil count 12.50 × 109/L (2.50–7.50), C-reactive protein 14 mg/L (<10), sodium 143 mmol/L (135–145), potassium 3.6 mmol/L (3.5–5.2), urea 4.8 micromol/L (2.5–7.5), creatinine 63 micromol/L (50–110), glucose 4.5 mmol/L (3.0–6.0), bilirubin 8 micromol/L (0–17), alkaline phosphatase 172 IU/L (100–280), alanine transaminase 25 IU/L (10–32), aspartate transaminase 114 IU/L (10–32), creatine kinase 146 IU/L (24–170), prothrombin time 13 s (9–13), and activated partial thromboplastin time 27 s (29–38).

## 4. Differential Diagnosis

Differential diagnosis that should be considered includes sepsis, the stiff man syndrome, heat stroke, delirium tremens, sympathomimetic and anticholinergic overdose, and neuroleptic malignant syndrome.

## 5. Treatment

Despite administering 15 mg of diazepam per rectum, her clonus persisted. The anaesthetic team was consulted and she was transferred to the intensive care unit (ITU) for intubation with concomitant infusions of propofol 1% at a rate of 1–20 mL/hour and alfentanil infusion 500 microgram/mL at a rate of 1–4 mL/hour. Despite these measures, clonus continued. Therefore, an atracurium infusion 500 mcg in 50 mL at a rate of 1–3 mL/hour was used with good effect. Whilst on the ITU, multiple blood, urine, and cerebrospinal fluid cultures were sent and subsequently reported as normal. A urine toxicology screen was also negative. Over the next 24 hours, the atracurium infusion was gradually weaned and she was extubated. She was reviewed by her psychiatry team and the entire episode was consistent with an unusual presentation of the serotonin syndrome. Antidepressant medications were discontinued and follow-up was arranged with the psychiatry team as an outpatient. Overall, she made a complete recovery and was discharged home two days later. To our knowledge she was not rechallenged with phenelzine.

## 6. Discussion

Serotonergic neurons are located in the midline raphe nuclei and play an integral part in the regulation of wakefulness, affective behaviour, food behaviour, thermoregulation, and motor tone. In the periphery, serotonin assists in the regulation of vascular tone and gastrointestinal motility [1, 2].

The serotonin syndrome can be a serious, life-threatening complication of serotonergic medications such as selective serotonin reuptake inhibitors, MAO inhibitors, and tricyclics. It arises when such agents increase serotonin neurotransmission at postsynaptic 5-hydroxytryptamine 1A and 5-hydroxytryptamine 2A receptors [3]. It usually occurs when two or more serotonin-modifying agents are used in combination; however, cases have been reported after single agent therapy with venlafaxine, moclobemide, sertraline, citalopram, and sumatriptan [4–8]. In this case monotherapy with phenelzine was most likely to be responsible given the patient's confirmed drug history.

The diagnosis of serotonin syndrome is clinical and there are no specific laboratory tests (see Table 1). Serum serotonin levels are not useful because it is the local concentration at the nerve terminal that is responsible for the presentation [9]. Thus, a strong clinical suspicion, exposure to serotonergic medications, demonstration of typical signs and symptoms, and exclusion of other possible medical and psychiatric disorders are required for diagnosis. Some nonspecific laboratory findings may be noted, including an elevated white blood cell count, elevated creatine phosphokinase, and decreased serum bicarbonate concentration [9].

The traditional diagnostic tool is the Sternbach criteria. More recently the Hunter Serotonin Toxicity Criteria have been developed and found to be more sensitive (84% versus 75%, resp.) and specific (97% versus 96%, resp.) than Sternbach criteria [10]. This patient met the Hunter criteria. The time of onset after initiation of therapy can range from minutes to days [11–18]. In one study 29 out of 39 patients with serotonin syndrome presented within 24 hours of medication initiation, overdose, or change in dosage, 5 out of 39 presented between 24 to 48 hours, and 5 out of 39 presented after 48 hours [9]. This latter observation partly supports our patient's presentation at two weeks after commencement of phenelzine.

Neuroleptic malignant syndrome (NMS) is perhaps the most frequently confused diagnostic consideration. NMS, however, typically presents after prolonged exposure to neuroleptics or withdrawal of dopamine agonists [19]. The onset is usually slow (days to weeks) and the physical examination is characterized by “lead-pipe” rigidity and extrapyramidal side effects but not myoclonus, hyperreflexia, or dilated pupils. Compared with serotonin syndrome, NMS is more commonly associated with high fevers, rhabdomyolysis, and mortality, 15–25% of all cases in one review [19]. Our patient did not have a history of neuroleptic medication usage; hence NMS was ruled out.

Common management pitfalls include failure to recognize serotonin syndrome, misdiagnosis, and failure to understand serotonin syndrome potentially rapid rate of progression [20]. Acute management of serotonin syndrome is based on two simple principles: discontinuation of all serotonergic medications and provision of necessary supportive care. Severe forms of the syndrome may require aggressive measures, including neuromuscular blocking agents, mechanical ventilation, benzodiazepines (for sedation), and external cooling [21].

In addition to supportive care and discontinuation of offending medications, there also may be a role for pharmacologic therapy in the acute management of serotonin syndrome. Specific agents have included benzodiazepines which help by reducing muscle rigidity and nonspecific serotonin receptor blockers such as cyproheptadine, chlorpromazine (can be given by intramuscular and intravenous routes), methysergide, and propranolol. Each of these agents has been credited with shortening the syndrome's duration [22] despite having no impact on mortality.

Serotonin syndrome often resolves within 24 hours of discontinuing the serotonergic agent and initiating care, but drugs with long half-lives or active metabolites may cause symptoms to persist [1]. Irreversible monoamine oxidase inhibitors (MAOIs) carry the greatest risk, and symptoms can persist for several days. Phenelzine is metabolised by acetylation; therefore it is possible that our patient was a slow acetylator which may have contributed to excess postsynaptic serotonin release [23]. Nevertheless, our patient showed complete resolution within 48 hours. Prognosis is generally favourable, as long as the entity is recognized and complications are treated appropriately.

## 7. Learning Points

- The serotonin syndrome is a well-known complication of treatment with serotonergic medications. Normally it occurs with combination therapy; however as this case demonstrates it can occur with monotherapy at therapeutic dosing.
- Presentation can be delayed as in this case.
- It is a self-limiting condition and resolves spontaneously with supportive care and discontinuation of culprit medications mostly in less than 24 hours.
- With early recognition and appropriate measures severe complications such as death of the patient can be avoided.

## Figures and Tables

**Table 1:** Key clinical features of the serotonin syndrome [21].

Cognitive/behavioural changes	Confusion, agitation, drowsiness, or coma.
Autonomic instability	Hyperthermia, hypertension, tachycardia, mydriasis, nausea, vomiting, diarrhoea, and diaphoresis.

Neuromuscular changes	Myoclonus, hyperreflexia, muscle rigidity, restlessness, tremor, positive Babinski, trismus, paraesthesia, and seizures.
